# Effects of antibiotics on the developing enamel in neonatal mice

**DOI:** 10.1007/s40368-021-00677-4

**Published:** 2021-10-29

**Authors:** A. J. Schmalfuss, A. Sehic, I. J. Brusevold

**Affiliations:** 1 Department of Clinical Dentistry, Faculty of Health Sciences, UiT Arctic University of Norway, Tromsö, Norway; 2 Institute of Oral Biology, University of Oslo Faculty of Dentistry, Oslo, Norway; 3 Institute of Clinical Dentistry, University of Oslo Faculty of Dentistry, Oslo, Norway

**Keywords:** Dental enamel hypoplasia, Molar-Incisor Hypomineralization, X-ray Microtomography, Tooth, Gentamicin, Ampicillin

## Abstract

**Purpose:**

Identifying factors causing Molar-Incisor Hypomineralization (MIH) is an ongoing challenge. Preterm infants, routinely treated with antibiotics in cases of suspected sepsis, are more commonly affected by dental developmental defects. This study aimed to investigate the effects of gentamycin and ampicillin on the developing enamel in neonatal CD-1 mice in vivo.

**Methods:**

Neonatal mice were randomized into a study (*n* = 36) and a control (*n* = 35) group. Antibiotics were injected intravenously for 4 days. All mice were sacrificed after 15–18 days. Micro-CT was used to analyse the mineral density (MD) of the enamel and the proportion of the enamel object volume (vol%) in first molars and incisors.

**Results:**

We demonstrated a significantly lower vol% enamel in the maxillary (30.9% vs. 32.7%; *p* = 0.004) and mandibular (32.5% vs. 34.6%; *p* = 0.015) molars in the study group than in the controls. The incisors were divided into segments upon analysis. We demonstrated both lower vol% and lower MD of the enamel in most segments in treated individuals compared to controls (*p* < 0.05).

**Conclusion:**

The reduced MD and vol% in the molars and incisors are likely to have been caused by the antibiotics given during tooth development. The presented analysis of teeth in neonatal mice with micro-CT could be a valid model for further research on dental developmental defects.

## Introduction

Dental enamel, covering the tooth crown, is produced by ameloblast cells. It is a unique tissue that exhibits remarkable wear and fracture resistance (Simmer et al. 2010). The aetiology of developmental enamel defects is multifactorial and provides challenges for basic science research and clinical management (Seow 2014). Molar-Incisor Hypomineralization (MIH) is one of the most common dental developmental disorders and affects one to four first permanent molars (FPMs). Permanent incisors are also frequently involved (Lygidakis et al. 2010). In Scandinavia, the prevalence of MIH varies from 13.9% in Northern Norway (Schmalfuss et al. 2016) to 37.3% in Denmark (Wogelius et al. 2008). Patients who are severely affected by MIH constitute a challenge in paediatric dentistry due to commonly occurring hypersensitive teeth and an increased risk of decay (de Souza et al. 2016). Repeated operative treatments may result in clinical behaviour management problems, dental fear and anxiety (Jalevik and Klingberg 2002). Our knowledge of the aetiology of MIH is still elusive. Locally disrupted amelogenesis has been suggested. Disturbances in tooth development must occur within the last trimester of pregnancy and up to the third year of a child’s life to result in MIH (Weerheijm and Mejare 2003). Several possible factors causing MIH have previously been reported (Garot et al. 2021). However, for most of them, the evidence is weak or absent. Environmental pollution, e.g., dioxins and polychlorinated biphenyls (Alaluusua et al. 1996), genetic predisposition (Jeremias et al. 2013) and several different conditions and diseases, including their treatments, have been suggested as aetiological factors. Several studies have associated a higher prevalence of MIH with serious illness during early childhood or complications, especially during the last trimester of pregnancy (Garot et al. 2021). A crucial question is whether enamel defects are caused by the illness itself, medications or a combination of both (Laisi et al. 2009).

The frequent use of antibiotics for the treatment of common childhood diseases, such as acute otitis media and respiratory infections, is potentially associated with MIH (Allazzam et al. 2014). Prematurity (< 38 weeks) (Mejia et al. 2019) or very low birthweight (LBW) (< 2500 g) (Ghanim et al. 2013) have also been associated with an increased occurrence of enamel defects in primary and permanent dentition. Brogardh-Roth et al. (2011) showed that MIH was more than twice as common in preterm infants and that LBW was a concomitant factor. These findings were confirmed by de Lima Mde et al. (2015). Furthermore, sepsis is one of the most common complications in premature infants. The recommendation by the World Health Organization for the treatment of preterm infants at risk for sepsis is followed by Norwegian hospitals, which administer ampicillin in combination with gentamicin (Fuchs et al. 2016). This treatment is given to the majority of preterm infants and has led to a significant reduction in mortality in premature and LBW infants (Bizzarro et al. 2005). Overprescription of antibiotics for fear of potentially dramatic consequences from a delayed neonatal sepsis diagnosis is also suspected based on empirical indications in many uninfected neonates (Fjalstad et al. 2016). Although the use of antibiotics during enamel formation in children has been associated with the aetiology of MIH (Serna et al. 2016), experimental evidence is scarce. The use of amoxicillin in early childhood has been associated with MIH (Laisi et al. 2009). An in vitro study demonstrated that amoxicillin affected enamel formation in cultured mouse embryotic tooth explants (Sahlberg et al. 2013). A recent in vivo study using relatively higher doses of amoxicillin showed a decrease in electron density in some rats, but the difference was not statistically significant (Feltrin-Souza et al. 2020).

To increase our understanding of how antibiotics other than amoxicillin affect enamel formation, ampicillin and gentamicin were administered to neonatal mice in doses similar to those given to LBW infants, and their effects on the enamel of molars and incisors were studied (Fuchs et al. 2016). The aim of this study was to determine whether the use of ampicillin and gentamicin in combination could cause mineralization disturbances in dental enamel.

## Materials and methods

### Animal model

Five pregnant mice (CD-1 strain) were maintained on a 12 h light–dark cycle at 21 °C and a relative humidity of 55%. The mice were given standard laboratory fodder and water ad libitum. The animals were kept according to the regulations of the Norwegian Gene Technology Act of 1994. In addition, they were kept in calm surroundings and an enriched environment facilitated with a variety of ‘toys’ to minimize suffering and distress. The experiment was approved by the Norwegian Local Veterinary Service (FOTS ID 8325). This study conforms to the ARRIVE guidelines.

From each litter, the neonatal mice (*n* = 71) were randomly allocated to a study group (*n* = 36) and a control group (*n* = 35). Based on the study by Sorensen et al. (2007) demonstrating that tail tip amputation only had minor short-term negative effects on mouse welfare, we performed this method to mark the mice of the control group. Starting at postnatal day 1 (P1) or 2 (P2), drugs (described later) were intravenously injected for 4 days in the morning as described by Glascock et al. (2011). Filtered green food dye (1:100) was added to the solutions to control access to the intravenous injection. The green dye rendered the drug distribution visible, as the normal pink colour of the neonates was converted to green. Before injection, the facial vein was made visible using a Wee Sight™ transilluminator (Philips, Amsterdam, Netherland). The solution was slowly injected with an insulin syringe (Micro-Fine™ 8 mm × 30 G; Becton Dickinson, New Jersey, USA) using a 2.5× magnifying visor loupe (Lactona, Bergen op Zoom, Netherland). Bleeding from the injection site was stopped by applying pressure with a gauze swab. After the transient stress from the injection was relieved, the mice were returned to the cage and kept under standard conditions. All treatments were performed by experienced, specially trained personnel, and all efforts were made to minimize suffering. The animals’ health and behaviour were monitored every day during the treatment period and every second day afterwards. According to the research protocol, all mice with health deficits or unnatural behaviour would have been euthanized immediately.

All neonatal mice were sacrificed by cervical dislocation according to the Norwegian Local Veterinary Service protocol at the age of P16 to P18. The jaws, including incisor and molar teeth, were dissected out and fixed in 70% ethanol. After fixation, the jaws were thoroughly cleaned by gentle brushing in running tap water using a stereomicroscope with a light source (SteREO Discovery. V8 & SteREO CL 1500 ECO; ZEISS, Oberkochen, Germany). Teeth with visible iatrogenic decay accidentally inflicted during preparation were excluded from the study.

All medicines were purchased from the hospital pharmacy (Sykehusapoteket Rikshospitalet, Oslo, Norway) and prepared for the study group as follows: the ampicillin solution was prepared by mixing powder and sterile physiological saline according to the manufacturer’s instructions (Pentrexyl; Bristol-Myers Squibb, Lysaker, Norway) and adding sterile filtered green food dye (1:100). Gentamicin (Gentamicin; B. Brown, Melsungen, Germany) was administered using a prefabricated solution (3 mg/ml) for injections. Before use, both antibiotic solutions were mixed according to the requested dose: ampicillin solution (200 mg/kg) 2.0 µl/g mouse body weight and gentamicin solution (4 mg/kg) 1.3 µl/g mouse body weight, equivalent to what is given to preterm infants in Norwegian hospitals. The injected volume per mouse was between 6.6 µl and 15.2 µl, corresponding to the body weight (2.0–4.6 g). For the control group, sterile saline was prepared with sterile filtered green food dye (1:100), and a volume corresponding to 3.3 µl/g mouse body weight was injected.

### Macro photography

As a part of the enamel examination, photographs were taken using a digital single lens reflex camera with a macro lens (D700 & AF Micro Nikkor 60 mm, 1:1; Nikon, Tokyo, Japan) and extension tubes (DG 12, 20 and 36 mm; Kenko Tokina, Tokyo, Japan) with standardized camera settings (ISO 200, f32, 3 s): the labial aspects of the upper and lower incisors and the occlusal surfaces of the first upper and lower molars. The photographs of 44 mice included in the study were projected onto a flat screen in a room with indirect, standardized lighting and examined individually and independently by three experienced dentists (ASc, ASe, IJB). The pictures were randomized and blinded before the examination. Enamel disturbances, such as demarcated opacities (white, yellow and brown colour), hypoplasias and other enamel defects were recorded. A joint score was decided for each recording, and consensus was reached through discussion when individual examiners differed.

### Micro-CT imaging

A micro-CT scanner (SkyScan 1172; Bruker, Brussels, Belgium) was used to provide three-dimensional images of mouse incisors and first molars to determine the enamel volume and density (de Lima Mde et al. 2015). The scanning protocol and parameters for human teeth described in previous studies (Johnsen et al. 2016) were used with adjustments to the requirements for analysis of murine dentitions. Samples were mounted vertically in customized tubes. Hydroxyapatite phantoms with known density were used to calibrate the instruments. The scanning parameters were 4.96 μm isotropic pixel size with a medium camera resolution and X-ray source (100 kV, 100 mA, 10 W) using a 0.5 mm Al filter. Samples were rotated 360° around their vertical axis, with a 0.4-rotation step and frame averaging of three. A flat field correction was performed before every scan. The X-ray attenuation coefficients were reconstructed with SkyScan software Nrecon (Bruker, Brussels, Belgium) to serial coronally oriented tomograms using a modified algorithm, with beam hardening of 20%, ring artefact correction of 12 and an attenuation coefficient range of 0.00–0.05. The greyscale was first defined using control mouse enamel, allowing the isolation of enamel alone without any other structure. The resulting histogram was used to determine a binary threshold of 60–255 for hard tissue, including enamel and dentine, and 130–255 for isolated enamel.

**Fig. 1:**
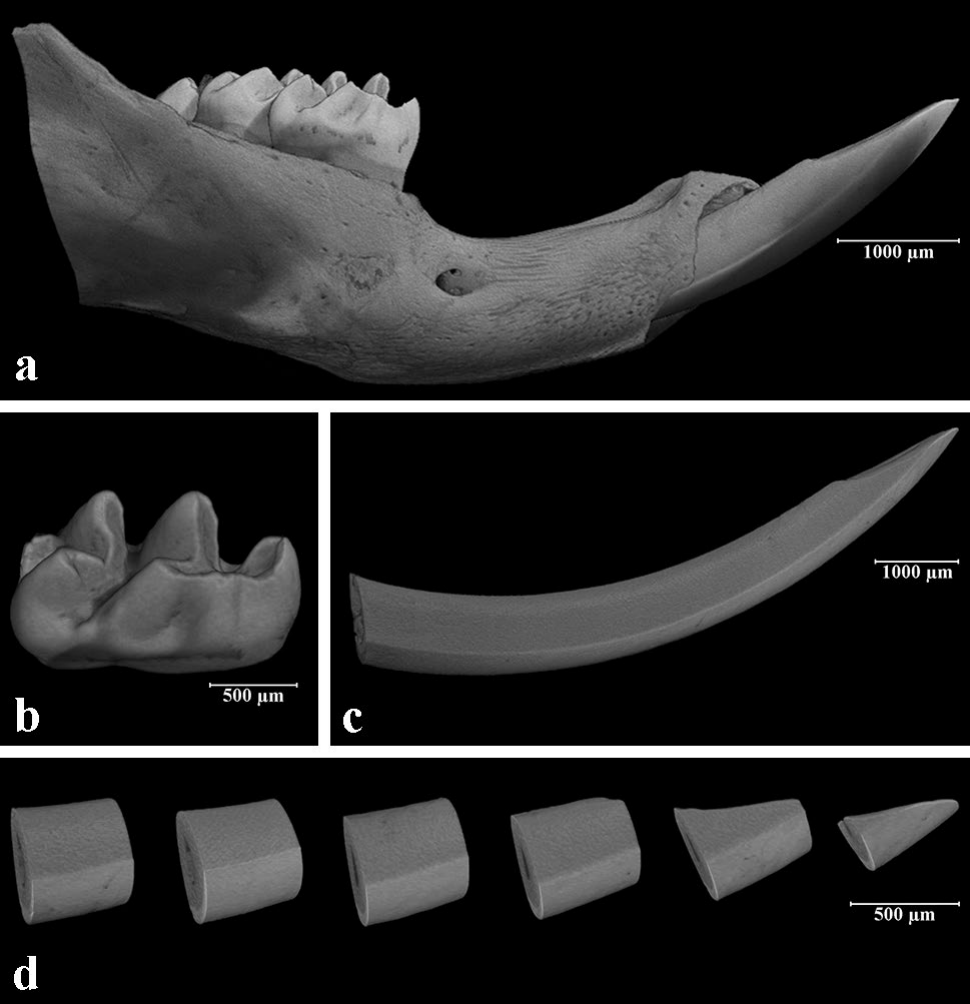
Three-dimensional reconstruction of slices from micro-CT of 18-day-old mice from the study group: **a** right mouse mandible, buccal view; **b** isolated crown of the first mandibular molar; **c** isolated crown of the mandibular incisor; **d** mandibular incisor divided into six segments (S1–S6)

Before analysing the enamel, first molars and incisors were isolated from the surrounding tissue prepared digitally using DataViewer (Bruker, Brussels, Belgium). The crown of the molars was isolated from the root at the cementoenamel junction in a standardized way (Fig. 1). The incisors were divided into six segments, each being 0.7 mm long starting at the incisal edge with S1 and ending at the most apical part of the tooth representing S6 following a standard protocol (Fig. 1). All images were inspected visually and with micro-CT, and iatrogenic artefacts were registered.

SkyScan software CTan and CTvol (Bruker, Brussels, Belgium) were used to determine enamel quality and quantity. The enamel was analysed according to mineral density (MD) (de Lima Mde et al. 2015) and the proportion of the enamel object volume (vol%) of the total hard dental tissue of the tooth or segment (Fig. 1).

### Statistical analysis

Results (enamel volume and density) are expressed as the mean ± SD of independent experiments in the upper and lower first molars and in every single segment (S1 to S6) of the incisors. Shapiro–Wilk’s test was used to test normal distribution. If normality failed, values were transformed using log10, √X or 1/X, and Shapiro–Wilk was repeated. One-way ANOVA was used to analyse the variance.

Welch’s *t* test was performed in cases, where both normality and variance failed. Student’s *t* test was used in cases with normal distribution and equal variance. The Mann–Whitney *U* test was performed when normality failed and the variance was equal. Groups were considered significantly different if *p* < 0.05. All calculations were carried out using SPSS version 24.0 (IBM SPSS Inc., Chicago, USA).

## Results

**Fig. 2:**
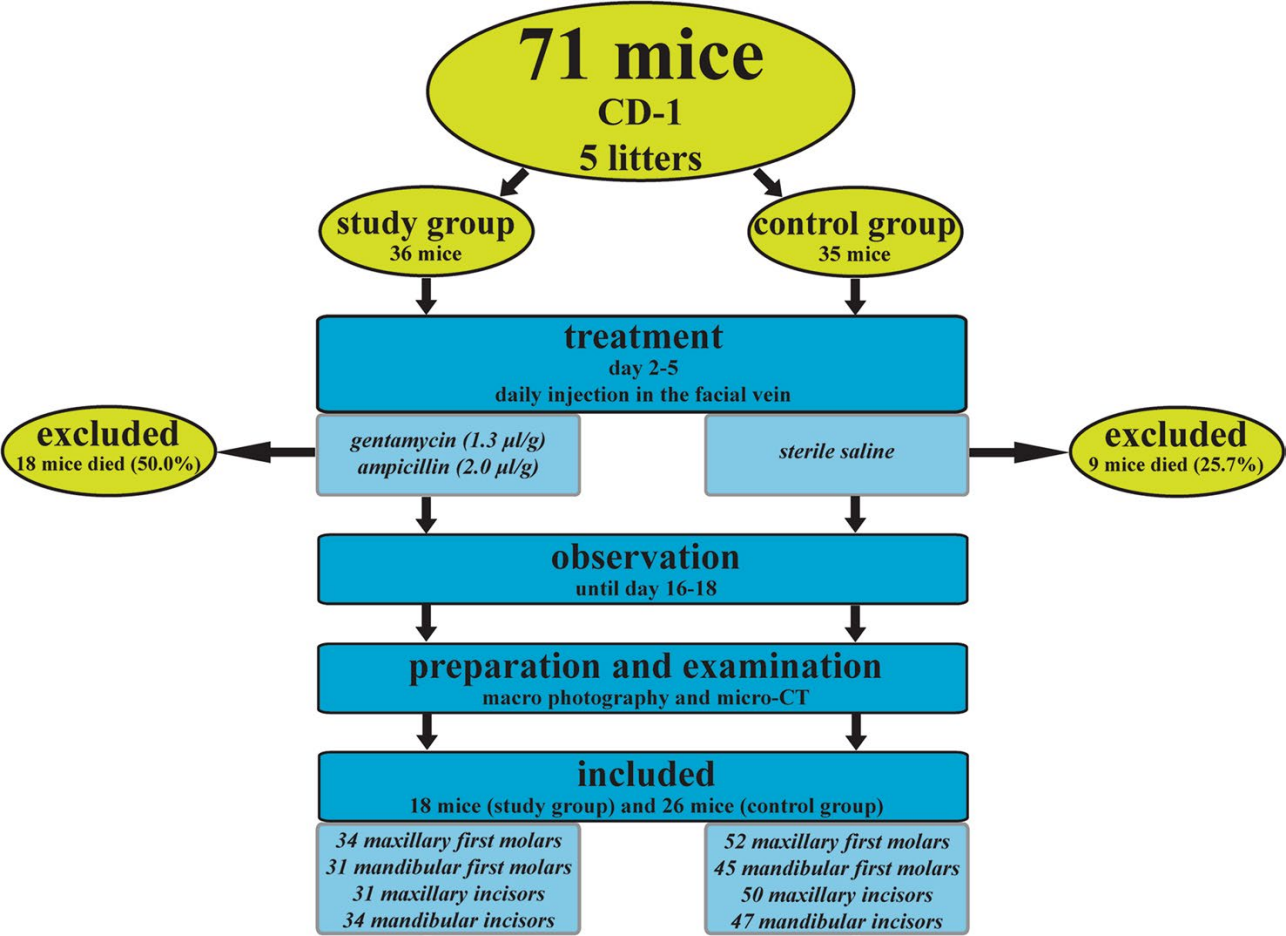
Flow chart of animals enrolled in the study

The flow chart of animals and teeth included in the final evaluation step of the study is presented in Fig. 2. None of the mice died directly under or immediately after the injection; however, during the observation period until P16 to P18, 18/36 mice in the study group and 9/35 mice in the control group died within P6 (Fig. 2). Unfortunately, these mice could not be examined due to natural animal cannibalism. The mean (SD) age of the mice at the final point was 16.9 (± 0.78) days. Among the included mice, 14 first molars and incisors (7.9%) were excluded due to iatrogenic decay. In total, 18 mice from the study group with 65 first molars and incisors were included. In the control group, 26 mice with 97 first molars and incisors were evaluated (Fig. 2).

**Fig. 3:**
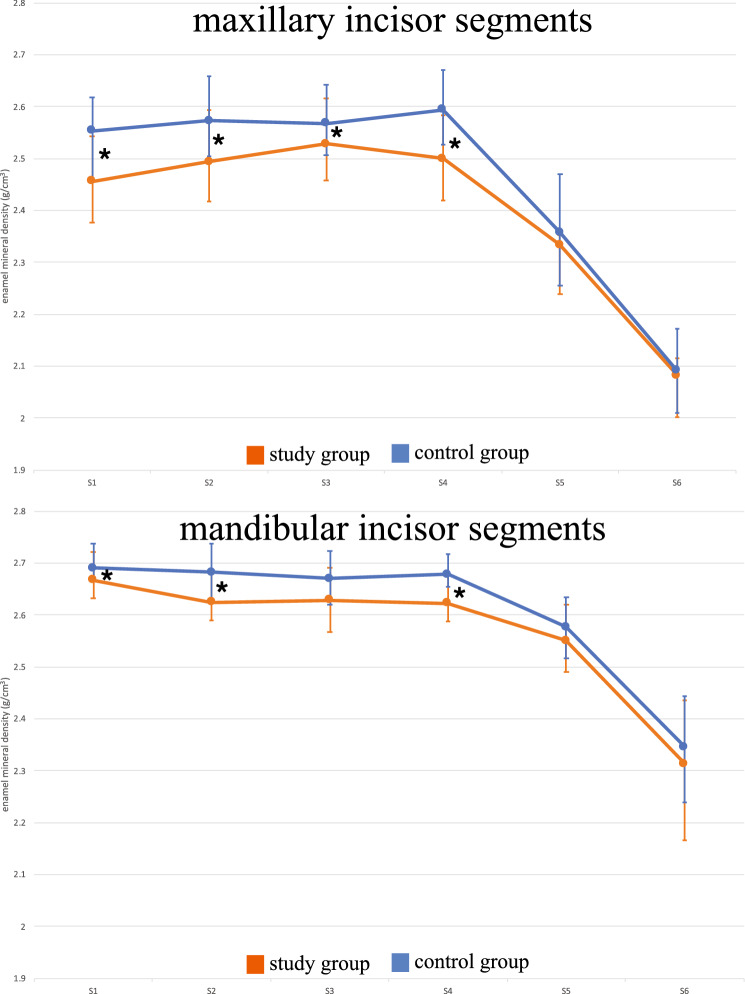
Median and interquartile range of the enamel mineral density (g/cm^3^) of maxillary and mandibular incisor segments (S1–S6); *significant difference between the study group and the control group (*p* < 0.05)

When dividing the incisors into six equal segments, the apical part of the enamel at approximately S5–S6 was less mineralized than the enamel closer to the incisal edge (Fig. 3). This made the enamel more vulnerable during the dissection process, and in two cases, the most apical segments (S6) were excluded because of enamel fractures.

**Fig. 4:**
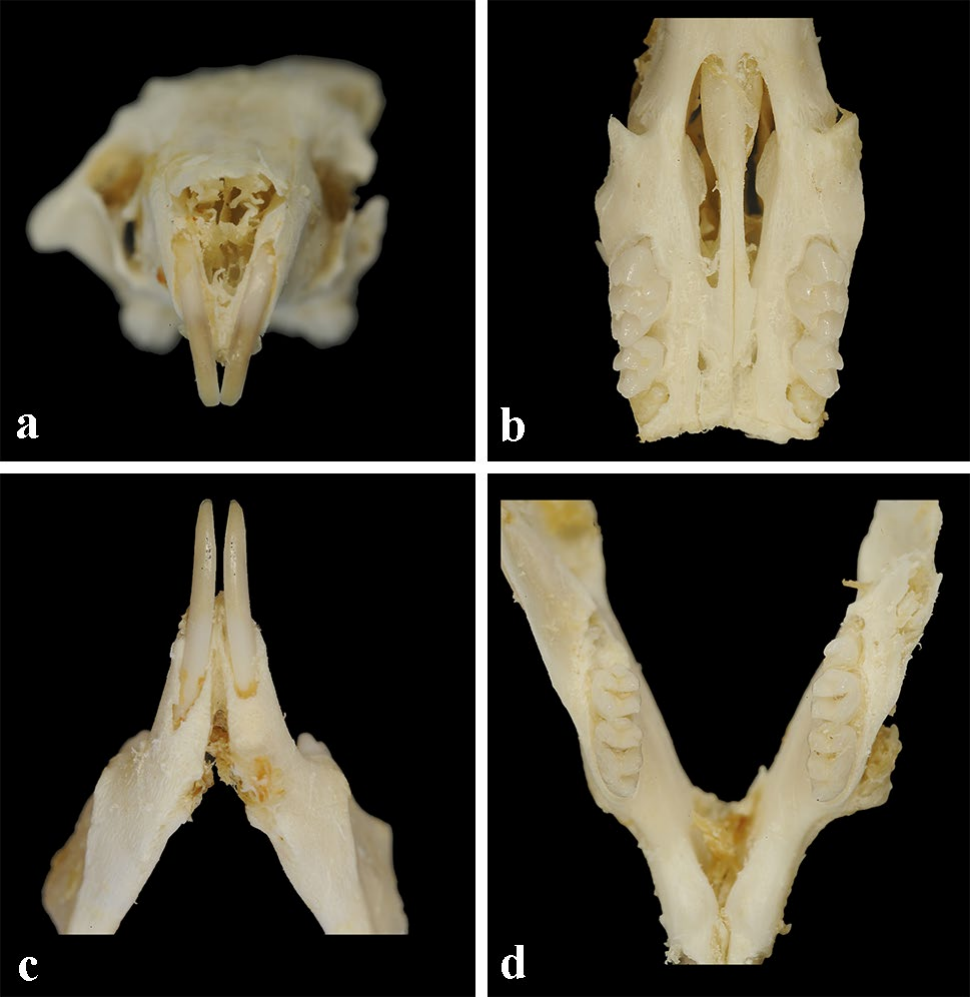
Photographs of teeth from mice in the study group: **a** maxillary mouse incisors; **b** maxillary mouse molars; **c** mandibular mouse incisors; **d** mandibular mouse molars

**Fig. 5:**
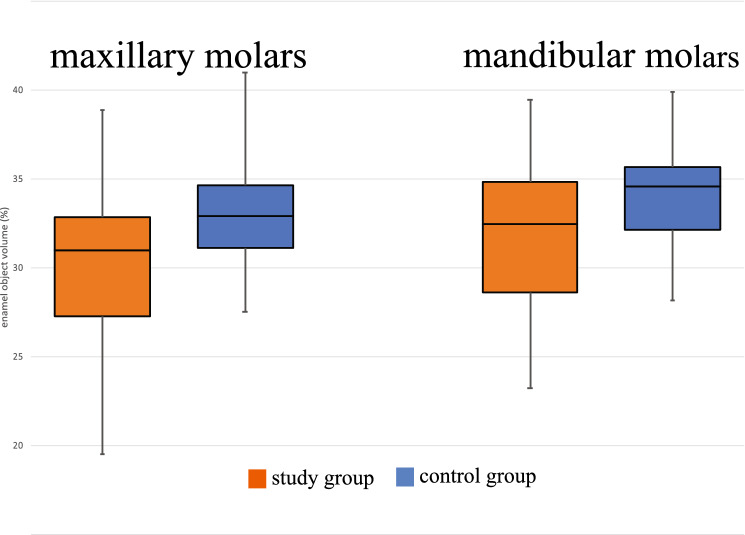
Enamel object volume (%) in the maxillary and mandibular first molars. There were significant differences between the study group and the control group in both the maxillary (*p* = 0.004) and mandibular teeth (*p* = 0.015)

**Fig. 6:**
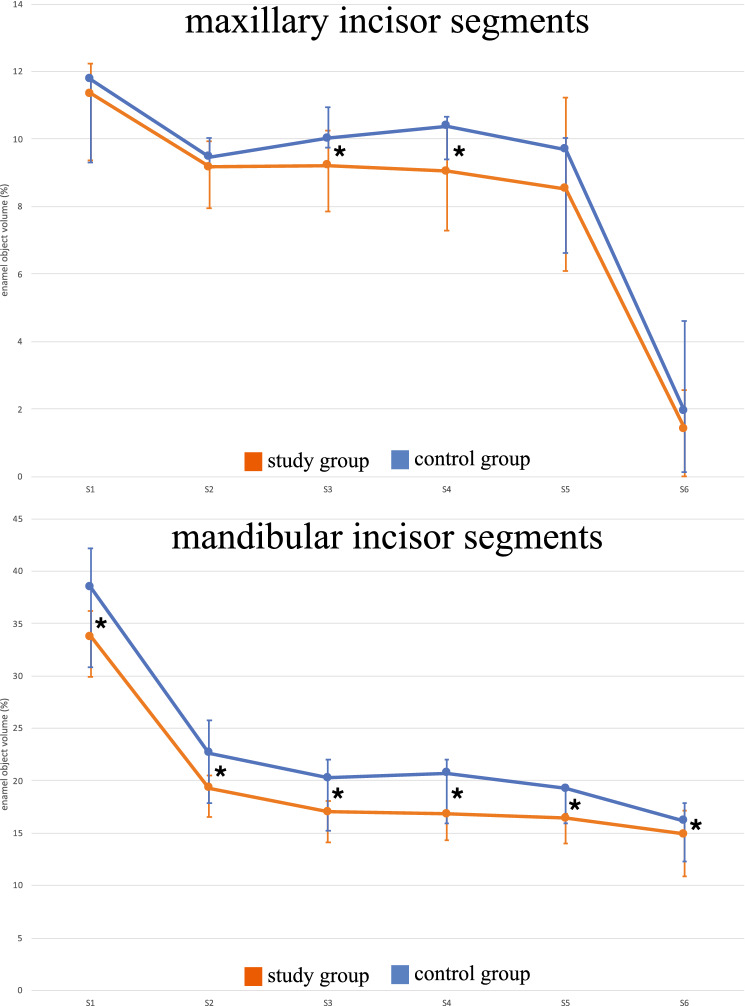
Median and interquartile range of the enamel object volume (%) in maxillary and mandibular incisor segments (S1–S6); *significant difference between the study group and the control group (*p* < 0.05)

No differences between the study group and the control group were observed by visual examination of macrophotographs (Fig. 4). The enamel vol% in the first molars was significantly lower in the study group than in the control group in both the upper (30.9% vs. 32.7%; *p* = 0.004) and lower (32.5% vs. 34.6%; *p* = 0.015) jaws (Fig. 5). The vol% of the study group was significantly lower in the mandibular incisors (*p* < 0.05) in all segments (S1–S6) of the mandibular incisors. In the maxilla, two incisor segments (S3, S4) showed a significantly lower vol% compared to the controls (Fig. 6). The MD of the enamel from the study group was significantly lower (*p* < 0.05) in four segments (S1–S4) from the maxillary incisors than that of the control group. In the mandibular incisors, the MD of the enamel was significantly lower in segments S1, S2, and S4 (Fig. 3). There was no significant difference between the MD of enamel from any of the first molars when comparing the study and the control groups.

## Discussion

Disturbances in enamel mineralization can affect both quality and quantity. In this study, we demonstrated that therapeutic doses of the antibiotics ampicillin and gentamicin reduced incisor MD values; similar alterations were observed in MIH. In contrast, the reduced enamel volume, which was demonstrated to indicate hypoplasia-like defects, is not consistent with MIH. In MIH-affected human molars, the MD values have been reported to be approximately 20% lower than those in clinically sound teeth, while the quantity of enamel was not affected (Fearne et al. 2004).

Studies on antibiotics and tooth development reveal conflicting results. Previous research using embryonic mouse molars in vitro showed that enamel was dose-dependently thicker in explants exposed to amoxicillin for 10 days (Laisi et al. 2009). The authors speculated that amoxicillin interfered with the function of ameloblasts by advancing the initiation of amelogenesis or the enamel accretion rate and concluded that this could explain the hypomineralization in MIH. Recently, temporarily significantly thinner enamel in rats treated pre- and postnatally with amoxicillin has been demonstrated by de Souza et al. (2016). Gottberg et al. (2014) showed that enamel thickness was not affected in rats treated prenatally with amoxicillin, but dose-dependent enamel hypomineralization was observed. This finding was confirmed in mice, and the authors concluded that chronic exposure to amoxicillin/clavulanic acid in doses used in humans caused hypomineralized enamel (Mihalas et al. 2016). Another study did not show any effect on enamel after amoxicillin treatment in rats (Kumazawa et al. 2012). It was suggested that amoxicillin affects the expression of the metalloproteinase MMP20, which has an important role in the degradation and removal of enamel proteins (Sahlberg et al. 2013). However, these findings were not confirmed by de Souza et al. (2016). In the present study, the antibiotics used were gentamicin and ampicillin administered intravenously, consistent with the treatment of human preterm infants diagnosed with sepsis. Due to filial infanticide and cannibalism, the cause of mortality in the present study remains unknown. It could be speculated that the medication used was close to the lethal dose. However, gentamicin, well known for its nephrotoxicity, has previously been used in mice at considerably higher doses without inducing toxic side effects (Blakley et al. 2008). It could also be speculated that the intravenous injection technique was too traumatic for some of the neonatal mice; however, care was taken to avoid injuring the mice. In other in vivo animal studies, antibiotics have been administered orally, intravenously, subcutaneously, intramuscularly and intraperitoneally. It was assumed that if partial paravenous or subcutaneous administration occurred, the dose was likely to be equally effective. Drug levels in blood serum or plasma were not measured to avoid additional burden on the animals. To the best of our knowledge, gentamicin and ampicillin have not yet been tested in isolation or in combination for their effect on enamel mineralization in vivo. In this study, the effects of these antibiotics were able to be examined in isolation without any confounding factors, such as infections seen in preterm infants treated with antibiotics.

In enamel research, micro-CT is a commonly accepted noninvasive technique to evaluate enamel density and volume representing hypomineralization and hypoplasia. In contrast to analyses with a significantly higher resolution, such as scanning electron microscopy (SEM) or quantitative backscattered electron (qBSE) imaging, micro-CT is not dependent on ground sections. Making accurate longitudinal ground sections of mouse mandibular incisors is difficult due to the small size and slight medio-lateral curvature of the teeth (Sidaly et al. 2015). While measurement of the enamel thickness in ground sections of teeth is limited to the selected slide, micro-CT enables a three-dimensional analysis of the enamel in the selected area (Fagrell et al. 2013). Enamel defects can be detected independently of their various tooth locations. In micro-CT, the enamel is detected and separated from dentine by its density. Severely hypomineralized enamel may be misinterpreted as dentine by micro-CT, leading to volume measurements of enamel being lower and those of dentine being higher. This bias was prevented by a visual examination of both clinical pictures and micro-CT images, where misinterpretations could have been registered (data not shown).

The difference in MD of enamel between the study and control groups was less than 4% in the present study. The entire section, i.e., both sound and affected enamel, was analysed. Due to the size of human molars, affected enamel can be analysed separately, which may result in greater differences (Fearne et al. 2004). The local temporary areas of hypomineralized enamel shown by Lyngstadaas et al. (1998) using SEM were not seen in the present study. It might be that micro-CT may not be sensitive enough to visualize these microscopic hypomineralizations.

Studies have shown that in first molars, ameloblasts at the tips of future cusps enter the secretory phase just after birth (P0), while at P2, crown mineralization advances by involving more ameloblasts in enamel production. On the fourth day (P4), dental crown morphogenesis is completed (Lungova et al. 2011). However, different strains of mice have been used in these studies, and differences in timing should be expected. Whereas Lungova et al. (2011) described a partially erupted first molar on day 16, all first molars were fully erupted in the present study. In the present study, medical treatment was performed from days P2 to P5; therefore, the maturation stage of enamel morphogenesis—but probably not the initial phase of amelogenesis—could be affected.

The eruption and enamel secretion rate of mandibular incisors (200 μm/d; 4.4 μm/d) is higher than that of maxillary incisors (140 μm/d; 7.3 μm/d) in adult mice (Sidaly et al. 2015). Slightly slower tooth development resulting in thinner enamel is expected in neonates (Schour and Massler 1967). In molars, secretion of enamel (7 μm/d) takes approximately 15 days with a final enamel thickness of approximately 100 μm (Lyngstadaas et al. 1998). In the present study, the enamel volume of the first molars was significantly reduced in the mice receiving drugs, which indicates that the secretory phase of amelogenesis is affected. This is consistent with results showing thinner enamel in rats treated with amoxicillin during the secretory stage of amelogenesis (de Souza et al. 2016). Smaller tooth size has been reported in preterm children and children with low body weight (< 2500 g) (Schuurs 2012). In human FPMs, the initiation of mineralization starts 1–7 weeks before birth (Antoine et al. 2009), while in mice, mineralization starts on day P2. This means that the onset of mineralization of FPMs in preterm infants is comparable to that in mice born to term.

Mice are convenient and extensively used for enamel research because of their comparable enamel structure. They are frequently used to examine the effect of medicine or environmental toxins on amelogenesis, but the potential for differences in pharmacokinetics between neonatal humans and mice remains uncertain (Warshawsky et al. 1981). In contrast to humans, mice have just a single dentition, probably the equivalent to human deciduous teeth (Tucker and Sharpe 2004). Although the present study aimed to explore MIH in permanent human dentition, similar defects exist in the primary dentition (Elfrink et al. 2012).

In contrast to incisors, mouse molars form a fast-developing model for odontogenesis in human molars. The mean formation time of the crown in human FPMs is more than 3 years (Reid and Dean 2006), while it is approximately 3 weeks in mice (Lungova et al. 2011). This quick sequence of stages in mice may make a direct comparison difficult (Fejerskov 1979), especially for studies of the transition stage (between secretion and maturation), which is assumed to be most vulnerable for the ameloblasts (Fearne et al. 2004). In the present study, mice were preferred to rats because of the slower eruption rate of the incisors, the longer enamel secretion phase and the greater enamel–dentine ratio in the former (Moinichen et al. 1996).

It is important to mention that the extrapolation of findings from the present animal study to humans must be conducted carefully. Rodent and human enamel has the same basic structural elements, prism and interprism; however, the spatial arrangement of prisms is considerably different (Warshawsky et al. 1981). Other differences include the speed at which enamel formation occurs and the incorporation of iron in the superficial enamel layer of rodent incisors (Risnes 1979). In the present study, we aimed to perform mouse injections with doses used in humans. However, the possible limitation of the present study may be that the drug uptake in rodents is different and/or that even the injection volumes/technique in such small animals may have varied. All these factors may have had an impact; however, we believe that this may not have significantly affected the major findings of our study.

## Conclusions

Considering the limitations of the present study, the following conclusions can be made: The intervention given to the neonatal mice in the present study was timed to influence the developing molars and incisors. The reduced MD and volume in the first molars and the erupted part of the incisors are likely to have been caused by antibiotics. The presented analysis of teeth in neonatal mice with micro-CT could be a valid model for further research on MIH. As the research on the effect of antibiotics on enamel development do not show conclusive results, further research is needed.

## Data Availability

All data are fully available without restriction. The data underlying the results presented in the study are available from the author Andreas Schmalfuss.

## References

[CR1] Alaluusua S, Lukinmaa PL, Vartiainen T, Partanen M, Torppa J, Tuomisto J (1996). Polychlorinated dibenzo-p-dioxins and dibenzofurans via mother’s milk may cause developmental defects in the child's teeth. Environ Toxicol Pharmacol.

[CR2] Allazzam SM, Alaki SM, El Meligy OA (2014). Molar incisor hypomineralization, prevalence, and etiology. Int J Dent.

[CR3] Antoine D, Hillson S, Dean MC (2009). The developmental clock of dental enamel: a test for the periodicity of prism cross-striations in modern humans and an evaluation of the most likely sources of error in histological studies of this kind. J Anat.

[CR4] Bizzarro MJ, Raskind C, Baltimore RS, Gallagher PG (2005). Seventy-five years of neonatal sepsis at Yale: 1928–2003. Pediatrics.

[CR5] Blakley BW, Hochman J, Wellman M, Gooi A, Hussain AE (2008). Differences in ototoxicity across species. J Otolaryngol Head Neck Surg.

[CR6] Brogardh-Roth S, Matsson L, Klingberg G (2011). Molar-Incisor Hypomineralization and oral hygiene in 10- to-12-yr-old Swedish children born preterm. Eur J Oral Sci.

[CR7] de Lima Mde D, Andrade MJ, Dantas-Neta NB, Andrade NS, Teixeira RJ, de Moura MS, de Deus Moura Lde F (2015). Epidemiologic study of Molar-Incisor Hypomineralization in schoolchildren in North-eastern Brazil. Pediatr Dent.

[CR8] de Souza JF, Gramasco M, Jeremias F, Santos-Pinto L, Giovanini AF, Cerri PS, Cordeiro RdCL (2016). Amoxicillin diminishes the thickness of the enamel matrix that is deposited during the secretory stage in rats. Int J Paediatr Dent.

[CR9] Elfrink ME, ten Cate JM, Jaddoe VW, Hofman A, Moll HA, Veerkamp JS (2012). Deciduous molar hypomineralization and molar incisor hypomineralization. J Dent Res.

[CR10] Fagrell TG, Salmon P, Melin L, Noren JG (2013). Onset of molar incisor hypomineralization (MIH). Swed Dent J.

[CR11] Fearne J, Anderson P, Davis GR (2004). 3D X-ray microscopic study of the extent of variations in enamel density in first permanent molars with idiopathic enamel hypomineralisation. Br Dent J.

[CR12] Fejerskov O (1979). Human dentition and experimental animals. J Dent Res.

[CR13] Feltrin-Souza J, Jeremias F, Alaluusua S, Sahlberg C, Santos-Pinto L, Jernvall J, Sova S, Cordeiro RCL, Cerri PS (2020). The effect of amoxicillin on dental enamel development in vivo. Braz Oral Res.

[CR14] Fjalstad JW, Stensvold HJ, Bergseng H, Simonsen GS, Salvesen B, Ronnestad AE, Klingenberg C (2016). Early-onset sepsis and antibiotic exposure in term infants: a nationwide population-based study in Norway. Pediatr Infect Dis J.

[CR15] Fuchs A, Bielicki J, Mathur S, Sharland M, Van Den Anker J. Antibiotic Use for Sepsis in Neonates and Children: 2016 Evidence Update. WHO Reviews. 2016.

[CR16] Garot E, Rouas P, Somani C, Taylor GD, Wong F, Lygidakis NA (2021). An update of the aetiological factors involved in molar incisor hypomineralisation (MIH): a systematic review and meta-analysis. Eur Arch Paediatr Dent.

[CR17] Ghanim A, Manton D, Bailey D, Marino R, Morgan M (2013). Risk factors in the occurrence of Molar-Incisor Hypomineralization amongst a group of Iraqi children. Int J Paediatr Dent.

[CR18] Glascock JJ, Osman EY, Coady TH, Rose FF, Shababi M, Lorson CL (2011). Delivery of therapeutic agents through intracerebroventricular (ICV) and intravenous (IV) injection in mice. J vis Exp.

[CR19] Gottberg B, Berne J, Quinonez B, Solorzano E (2014). Prenatal effects by exposing to amoxicillin on dental enamel in Wistar rats. Med Oral Patol Oral Cir Bucal.

[CR20] Jalevik B, Klingberg GA (2002). Dental treatment, dental fear and behaviour management problems in children with severe enamel hypomineralization of their permanent first molars. Int J Paediatr Dent.

[CR21] Jeremias F, Koruyucu M, Kuchler EC, Bayram M, Tuna EB, Deeley K, Pierri RA, Souza JF, Fragelli CM, Paschoal MA, Gencay K, Seymen F, Caminaga RM, dos Santos-Pinto L, Vieira AR (2013). Genes expressed in dental enamel development are associated with Molar-Incisor Hypomineralization. Arch Oral Biol.

[CR22] Johnsen GF, Sundnes J, Wengenroth J, Haugen HJ (2016). Methodology for morphometric analysis of modern human contralateral premolars. J Comput Assist Tomogr.

[CR23] Kumazawa K, Sawada T, Yanagisawa T, Shintani S (2012). Effect of single-dose amoxicillin on rat incisor odontogenesis: a morphological study. Clin Oral Investig.

[CR24] Laisi S, Ess A, Sahlberg C, Arvio P, Lukinmaa PL, Alaluusua S (2009). Amoxicillin may cause molar incisor hypomineralization. J Dent Res.

[CR25] Lungova V, Radlanski RJ, Tucker AS, Renz H, Misek I, Matalova E (2011). Tooth-bone morphogenesis during postnatal stages of mouse first molar development. J Anat.

[CR26] Lygidakis NA, Wong F, Jalevik B, Vierrou AM, Alaluusua S, Espelid I (2010). Best Clinical Practice Guidance for clinicians dealing with children presenting with Molar-Incisor -Hypomineralisation (MIH): an EAPD Policy Document. Eur Arch Paediatr Dent.

[CR27] Lyngstadaas SP, Moinichen CB, Risnes S (1998). Crown morphology, enamel distribution, and enamel structure in mouse molars. Anat Rec.

[CR28] Mejia JD, Restrepo M, Gonzalez S, Alvarez LG, Santos-Pinto L, Escobar A (2019). Molar incisor hypomineralization in colombia: prevalence, severity and associated risk factors. J Clin Pediatr Dent.

[CR29] Mihalas E, Matricala L, Chelmus A, Ghetu N, Petcu A, Pasca S (2016). The role of chronic exposure to amoxicillin/clavulanic acid on the developmental enamel defects in mice. Toxicol Pathol.

[CR30] Moinichen CB, Lyngstadaas SP, Risnes S (1996). Morphological characteristics of mouse incisor enamel. J Anat.

[CR31] Reid DJ, Dean MC (2006). Variation in modern human enamel formation times. J Hum Evol.

[CR32] Risnes S (1979). A method of calculating the speed of movement of ameloblasts during rat incisor amelogenesis. Arch Oral Biol.

[CR33] Sahlberg C, Pavlic A, Ess A, Lukinmaa PL, Salmela E, Alaluusua S (2013). Combined effect of amoxicillin and sodium fluoride on the structure of developing mouse enamel in vitro. Arch Oral Biol.

[CR34] Schmalfuss A, Stenhagen KR, Tveit AB, Crossner CG, Espelid I (2016). Canines are affected in 16-year-olds with Molar-Incisor hypomineralisation (MIH): an epidemiological study based on the Tromso study: “Fit Futures”. Eur Arch Paediatr Dent.

[CR35] Schour I, Massler M, Griffith JQ, Farris EJ (2021). The teeth. The rat in laboratory investigation.

[CR36] Schuurs A (2012). Pathology of the hard dental tissues.

[CR37] Seow WK (2014). Developmental defects of enamel and dentine: challenges for basic science research and clinical management. Aust Dent J.

[CR38] Serna C, Vicente A, Finke C, Ortiz AJ (2016). Drugs related to the etiology of molar incisor hypomineralization: a systematic review. J Am Dent Assoc.

[CR39] Sidaly R, Risnes S, Khan QE, Stiris T, Sehic A (2015). The effect of hypoxia on the formation of mouse incisor enamel. Arch Oral Biol.

[CR40] Simmer JP, Papagerakis P, Smith CE, Fisher DC, Rountrey AN, Zheng L, Hu JC (2010). Regulation of dental enamel shape and hardness. J Dent Res.

[CR41] Sorensen DB, Stub C, Jensen HE, Ritskes-Hoitinga M, Hjorth P, Ottesen JL, Hansen AK (2007). The impact of tail tip amputation and ink tattoo on C57BL/6JBomTac mice. Lab Anim.

[CR42] Tucker A, Sharpe P (2004). The cutting-edge of mammalian development; how the embryo makes teeth. Nat Rev Genet.

[CR43] Warshawsky H, Josephsen K, Thylstrup A, Fejerskov O (1981). The development of enamel structure in rat incisors as compared to the teeth of monkey and man. Anat Rec.

[CR44] Weerheijm KL, Mejare I (2003). Molar incisor hypomineralization: a questionnaire inventory of its occurrence in member countries of the European Academy of Paediatric Dentistry (EAPD). Int J Paediatr Dent.

[CR45] Wogelius P, Haubek D, Poulsen S (2008). Prevalence and distribution of demarcated opacities in permanent 1st molars and incisors in 6 to 8-year-old Danish children. Acta Odontol Scand.

